# Supplementary material for: Digital and Navigational Health Literacy in Surgical Patients: Vulnerabilities in the Transition to Post-Discharge Care
Source: Healthcare (Basel). 2025 Dec 10;13(24):3227. doi: 10.3390/healthcare13243227 (PMC12732921; doi:10.3390/healthcare13243227)
Supplement: Supplementary file 1 [file healthcare-13-03227-s001.zip › healthcare-4000999-supplementary.pdf]

HLS_{19}-NAV-PT

Please cite as: Directorate-General of Health for HLS_{19} (2020): HLS_{19}-NAV-PT_Portuguese – The Portuguese instrument for measuring navigational health literacy in the general population. M-POHL. Lisbon

1. NAVEGAÇÃO

NÃO INTERESSA SE USA A INFORMAÇÃO PARA SI OU PARA OS OUTROS. POR "SERVIÇO DE SAÚDE" ENTENDE-SE MÉDICO, ESPECIALISTA, HOSPITAL, LAR DE IDOSOS, INSTITUIÇÕES DE REABILITAÇÃO OU SAÚDE MENTAL

**Q33. Quão fácil ou difícil é...**	Muito fácil	Fácil	Difícil	Muito difícil	Não sabe
...perceber a informação sobre como funciona o sistema de saúde? *[e.g. tipo de serviços de saúde disponíveis]*					
...determinar qual o tipo de serviços de saúde que precisa em caso de problema de saúde?					
...determinar em que medida o seu seguro de saúde cobre determinado serviço de saúde? *[e.g. se existem comparticipações]*					
...compreender a informação sobre as reformas contínuas aos cuidados de saúde que podem afetar os seus cuidados de saúde?					
...saber quais os seus direitos como doente ou utente do sistema de saúde?					
...decidir-se por determinado serviço de saúde? *[e.g. escolher entre diferentes hospitais]*					
...encontrar informação sobre a qualidade de determinado serviço de saúde?					
...avaliar se determinado serviço de saúde irá satisfazer as suas expectativas e pretensões em termos de cuidados de saúde?					
...compreender como fazer uma marcação em determinado serviço de saúde?					

...encontrar apoios que o(a) possam ajudar a orientar-se no sistema de saúde?					
...localizar, numa instituição de saúde, a pessoa certa a contactar no seu caso específico?<br>*[e.g. num hospital]*					
...fazer valer os seus direitos, caso os cuidados de saúde não satisfaçam as suas necessidades?					
